# Oligosaccharides production from coprophilous fungi: An emerging functional food with potential health-promoting properties

**DOI:** 10.1016/j.btre.2022.e00702

**Published:** 2022-01-21

**Authors:** Jeff Ojwach, Adegoke Isiaka Adetunji, Taurai Mutanda, Samson Mukaratirwa

**Affiliations:** a School of Environmental Sciences, University of East Anglia, Norwich Research Park, Norwich NR4 7TJ, United Kingdom; b Department of Biodiversity and Conservation Biology, Faculty of Natural Science, University of the Western Cape, Private Bag X17 Bellville 7530, South Africa; c School of Life Sciences, College of Agriculture Engineering and Science, University of KwaZulu-Natal (Westville Campus), Private Bag X54001, Durban 4000, South Africa; d Centre for Algal Biotechnology, Department of Nature Conservation, Faculty of Natural Sciences, Mangosuthu University of Technology, P.O. Box 12363, Jacobs 4026, Durban, South Africa; e One Health Center for Zoonoses and Tropical Veterinary Medicine, Ross University, School of Veterinary Medicine, P.O. Box 334, Basseterre, St. Kitts, West Indies

**Keywords:** FOS, Fructooligosaccharide, IOS, Inulooligosaccharide, Ftase, Fructosyltransferase, Ffase, β-fructofuranosidase, GOS, Galactooligosaccharide, IMO, Isomaltooligosacharide, MOS, Maltooligosaccharide, XOS, Xylooligosaccharide, Oligosaccharides, Fructooligosaccharides, Inulooligosaccharides, Inulinase, Fructosyltransferase, Coprophilous fungi

## Abstract

Functional foods are essential food products that possess health-promoting properties for the treatment of infectious diseases. In addition, they provide energy and nutrients, which are required for growth and survival. They occur as prebiotics or dietary supplements, including oligosaccharides, processed foods, and herbal products. However, oligosaccharides are more efficiently recognized and utilized, as they play a fundamental role as functional ingredients with great potential to improve health in comparison to other dietary supplements. They are low molecular weight carbohydrates with a low degree of polymerization. They occur as fructooligosaccharide (FOS), inulooligosaccharadie (IOS), and xylooligosaccahride (XOS), depending on their monosaccharide units. Oligosaccharides are produced by acid or chemical hydrolysis. However, this technique is liable to several drawbacks, including inulin precipitation, high processing temperature, low yields, and high production costs. As a consequence, the application of microbial enzymes for oligosaccharide production is recognized as a promising strategy. Microbial enzymatic production of FOS and IOS occurs by submerged or solid-state fermentation in the presence of suitable substrates (sucrose, inulin) and catalyzed by fructosyltransferases and inulinases. Incorporation of FOS and IOS enriches the rheological and physiological characteristics of foods. They are used as low cariogenic sugar substitutes, suitable for diabetics, and as prebiotics, probiotics and nutraceutical compounds. In addition, these oligosaccharides are employed as anticancer, antioxidant agents and aid in mineral absorption, lipid metabolism, immune regulation etc. This review, therefore, focuses on the occurrence, physico-chemical characteristics, and microbial enzymatic synthesis of FOS and IOS from coprophilous fungi. In addition, the potential health benefits of these oligosaccharides were discussed in detail.

## Highlights

• Coprophilous fungi biodiversity provides fructosyltransferase (Ftase) and inulinase enzymes for the synthesis of oligofructans.
• Microbial enzymes as unique bioresources for oligomerization.
• Oligosaccharides occur in different forms, depending on their monosaccharide units.
• Recent findings on the production and application of short-chain oligosaccharides from coprophilous fungi.
• Oligosaccharides from coprophilous fungi possess potential health benefits.

## Graphical abstract

**Figure:**
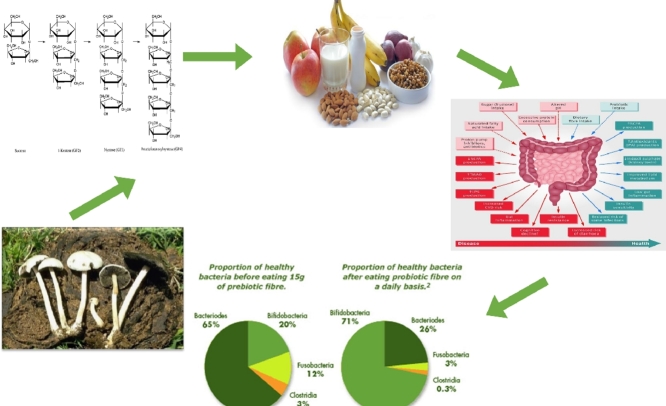

## 1 Introduction

The design of food products that confer health-promoting properties is emerging and there is a growing acceptance that functional food can lead to disease prevention, well-being, and treatment [1]. Ideally, all food can be said to be functional if they contain components that provide energy and nutrients necessary for growth and survival [2]. Due to advances and desires in food technology and the emerging scientific evidence linking diet to disease, there is a need to address the consumption of functional foods with health-promoting properties besides basic nutrition [3]. Food supplements with health-promoting properties help in gut manipulation and composition towards a salutary regimen [4]. Most soluble fibers do not contribute to fecal bulking, but are fermented by the gut bacteria and thus give rise to metabolites such as short-chain fatty acids (SCFAs) by increasing the proliferation of endogenous *Bifidobacterium* and *Lactobacillus* composition, thereby creating a prebiotic effect [5].

Prebiotics are non-digestible food ingredients (including polysaccharides and oligosaccharides) that affect the host by selective stimulation of growth and/or of one or a limited number of bacteria in the gut and thus improve health [6]. Prebiotic therapies have been recognized for the treatment of gut-related illnesses such as relief of constipation, insulin resistance, diarrhea suppression, obesity, and some cardiovascular diseases associated with dyslipidemia [7]. For a food ingredient to be considered as a prebiotic, it must resist gastric metabolism and hydrolysis from enzymatic activity [5, 8, 9]. Secondly, the oligomers must be fermented by intestinal microbes and also stimulate the activity of selective bacteria in the colon [10].

In addition to the prebiotic effect, these food ingredients are still important due to their nutraceutical effects by possessing health or medical benefits including prevention or treatment of diseases [11]. Such products include dietary supplements such as oligosaccharides, isolated nutrients, specific diets, genetically engineered foods, herbal products, and processed foods [12], [13], [14]. Specifically, these food products include oligosaccharides, which are dietary carbohydrates and play a fundamental role as functional ingredients when compared to probiotics, sugars, polyunsaturated fatty acids, and peptides. The requisite end products of carbohydrates metabolism are short-chain fatty acids. These include butyric acid, acetic acid, and propionic acid, which are used up by host organisms as a source of energy [15].

Microbes are also documented widely as an alternative source of oligosaccharide production [16], [17], [18], [19]. Oligosaccharides are sugar combinations with the degree of polymerization (DP_3_ to DP_10_), and are from plant inulin or produced commercially from sucrose as substrate [20]. In the first approach, inulin is cleaved from chicory randomly by microbial endoinulinase (EC 3.2.1.7), yielding oligofructosides [21]. In the second approach, sucrose is fructosylated to GF_2_, GF_3,_ and GF_4_ by β-fructofrunosidases (EC 3.2.1.26) or *β*-fructosyltransferases (EC 2.4.1.100) from fungal genera including *Aureobasidium* and *Aspergillus* [22, 23].

A combination of probiotics and prebiotics are used together to take advantage of synergic effects in food application and biotechnology and the mixture is called synbiotic [30]. The health effects of functional foods, including their nutraceutical effect, have led to numerous studies on food-grade oligosaccharides which include fructooligosaccharides (FOS), inulooligosaccharides (IOS), xylooligosaccharides (XOS), galactooligosaccharides (GOS), mannooligosaccharide (MOS) amongst classes of prebiotics [31], [32], [33], [34], [35], [36]. To produce food-based FOS and IOS, microbial enzymatic synthesis remains an attractive and desirable approach, as it is environment friendly, emits fewer emissions and by-products, and operates at low temperatures [37]. The present review focuses on the occurrence and microbial enzymatic production of FOS and IOS from new coprophilous fungi. Thereafter, the potential health benefits of the oligosaccharides were discussed explicitly.

## 2 Coprophilous fungi-Habitats and occurrence

**Fig. 1:**
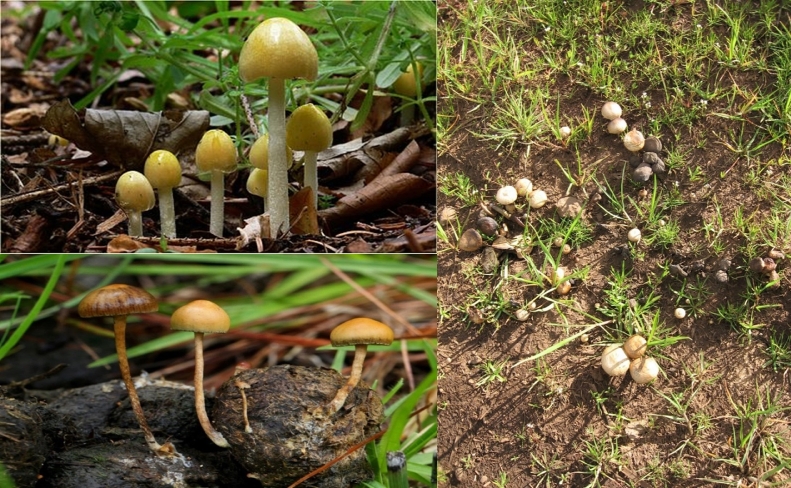
Coprophilous fungi growing on herbivore dung substrata.

Coprophilous fungi, also known as fimicolous species are dung-loving fungi, found on dung substratum [38, 39]. They are a group of saprophytic fungi adapted to life on dung and fecal pellets of herbivores (Fig. 1) [40]. These fungi rely on terrestrial warm-blooded herbivores to complete their life cycle [41]. When herbivores graze on vegetation, they ingest spores from coprophilous and non-coprophilous fungi along with vegetation [42]. The spores of non-coprophilous fungi are killed by high temperatures and gastric juices in the gastrointestinal tract of the herbivores while coprophilous fungal spores survive in the gut, undergo hydrolysis, and are passed out to germinate, grow and fruit on dung [43]. However, any dung can yield fungi, but herbivore dung has been regarded as the best source of coprophilous fungi. Moreover, several investigations involving herbivore dung have demonstrated potential for enzyme production for industrial and biotechnological applications (Table 1). This fungus has a cosmopolitan distribution, as they occur in many herbivore species around the world [44, 45].

Coprophilous fungi are classified into different morphological keys: key one (MJR) belongs to coprophilous ascomycetes that are a very diverse group with many species yet to be discovered [46]. The second key includes the original plectomycete key (RW), which contains fungi that are not biased on herbivore dung but occur in horn, hair, and cadavers as well as on carnivore dung [46]. The third key (RW, p52) belongs to basidiomycetes of dung-associated debris. The fourth key (MJR, p63) includes zygomycetes, found to appear first on freshly dropped dung, but which soon disappear [46].

Herbivore dung is a rich substratum of coprophilous fungi and supports high species diversity. Fruiting bodies of dung fungi appear in succession mostly following the sequence: Zygomycotina, Ascomycotina, and Basidiomycotina [42]. Dung fungi play a vital role in the mineralization and decomposition of herbivore dung while, some display few modifications peculiar to their habitat [42, 47].

### 2.1 Potential of coprophilous fungi in oligosaccharide production

Fungi that grow on herbivore dung are full of fiber from dung biomass and have potential cellulolytic activity [48]. Cellulose is a linear glucose polymer linked by β−1,4-glycosidic bond, forming a large component of plant biomass [38]. Herbivore dung contains high amounts of readily available complex carbohydrates, made up of cellulose, hemicellulose, pectin, lignin, and high nitrogen content. In addition, they have a high moisture content, vitamin, growth factors, and minerals [40, 47]. The ruminal ecosystem represents the most potent fibrolytic fermentation system known. It is composed of a diverse population of obligate anaerobic fungi, bacteria, and protozoa [49]. Coprophilous fungi in the rumen produce potent fibrolytic enzymes that can degrade recalcitrant plant polymers [48]. The gut metabolism of herbivores is specifically adapted for highly specialized microbial processing of complex plant polysaccharides ingested [49]. Since dung is egested with plant material, cells, and interwoven matrix of plant polymers from the herbivore rumen due to their incomplete digestion and consequently microbes on dung use them up. The array of enzymes in the rumen is not only from gut microbial diversity but also from the multiplicity of fibrolytic enzymes produced by individual microbes [49].

Recently, from our laboratory, sixty-one autochthonous coprophilous fungal strains were screened for the ability to biotransform sucrose and inulin into FOS and IOS by producing fructosyltransferase and inulinase, respectively. The isolates exhibited high transfructosylating activity and produced short-chain FOSs including GF_3_, GF_4,_ and GF_5_. Coprophilous fungus isolate XOPB-48 identified as *Aspergillus niger* showed a robust combination of high extracellular transferase activity following HPLC-RI analysis [50]. The enzyme exhibited a good transfructosylating activity by catalyzing sucrose to FOS with an I/S ratio of 1.77. The utilization of herbivore dung as a cheap and readily available bioresource raw material allows the development of low-cost bioprocess for FOS and IOS production. In addition, the complex carbohydrate and bioactive characteristics of cellulose and lignin in dung biomass display an unexplored reservoir for novel enzymes as they can produce enzymes with transfructosylating activity.

## 3 Oligosaccharides

Oligosaccharides form part of new functional food with great potential to improve health due to their physicochemical characteristics [51]. They are classified as glycosides since they contain 3–10 sugars moieties [52]. Oligosaccharides are carbohydrates with low molecular weight and low DP [51]. Carbohydrates are the main group that forms oligosaccharides; their monosaccharide units include glucose, galactose, fructose, and xylose. The non-digestible oligosaccharides emanate from the survey that carbon atoms of the monosaccharides have some disposition that make osidic bonds non-digestible to hydrolytic activity of enzymes in the human intestine [53]. Oligosaccharide stability differs according to classes depending on sugar residues present and anomeric configuration [54, 55]. They also have high moisture retaining capability, preventing excessive drying, and low water activity that inhibits microbial contamination [56].

### 3.1 Physicochemical and functional properties of oligosaccharides

Oligosaccharides have biofunctional and physicochemical properties that make them desirable for consumption as food ingredients or supplements [51]. Incorporation of oligosaccharides enriches the rheological and physiological characteristics of foods [57]. This is predominantly due to their water solubility and sweetness. Oligosaccharides are slightly sweeter than sucrose (0.3–0.6 times), but their sweetness is dependent on their degree of polymerization, chemical array, and level of mono- and disaccharide present in the mixture [56]. The viscosity of fructooligosaccharide (FOS) solution is relatively higher than that of mono- and disaccharide (sucrose) at the same concentration [31]. They are more viscous due to their higher molecular weight [58]. They alter the amount of browning in food by recasting the freezing temperature of some foods. They control microbial contamination by absorbing water since they act as a drying agent due to their moisture-retaining capability [59]. FOSs have higher thermal stability than sucrose; they are stable within the normal pH range of foods (pH 4.0–7.0) [27]. Their stability is dependent on ring form, sugar residue content, anomeric configuration, and linkage type.

Oligosaccharides are used as low cariogenic sugar substitutes, as they are inactivated by mouth enzymes or in the upper gastrointestinal tract to form acid or polyglucans due to their physicochemical characteristics of being less sweet, making them suitable for consumption by diabetics [60, 61]. They show immoderately high structural diversity than oligonucleotides and oligopeptides [62].

### 3.2 Occurrence of fructooligosaccharides

**Fig. 2:**
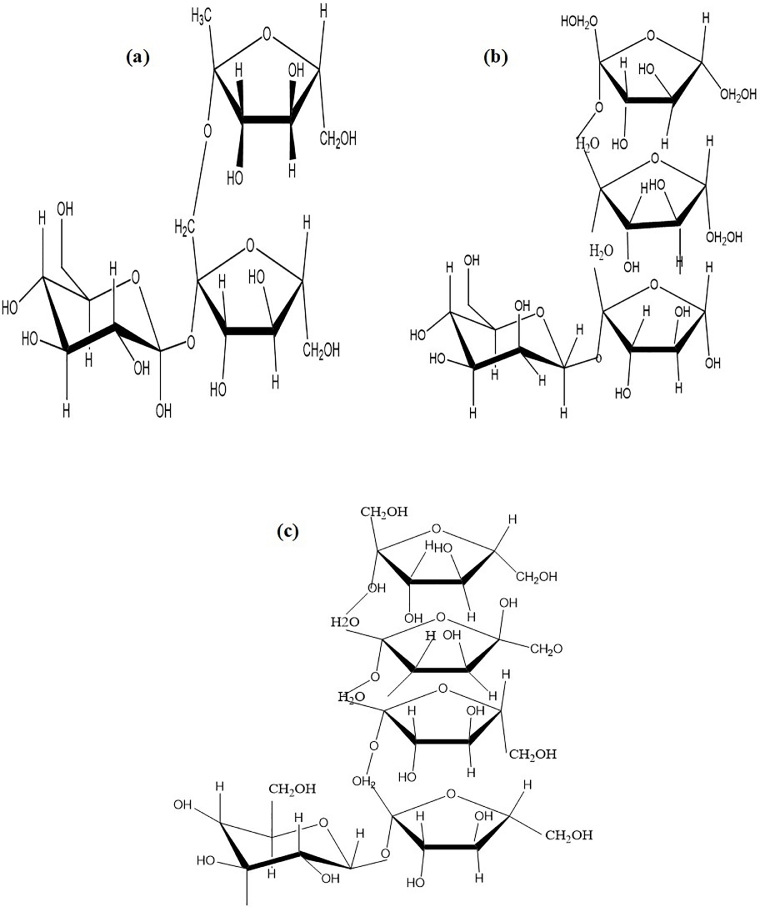
The structural composition of the main constituent of FOS (a) 1-kestose (GF_2_), (b) 1-nystose (GF_3),_ and (c) fructofuranosyl nystose (GF_4_) Adopted from (Dominguez et al., 2014).

**Fig. 3:**
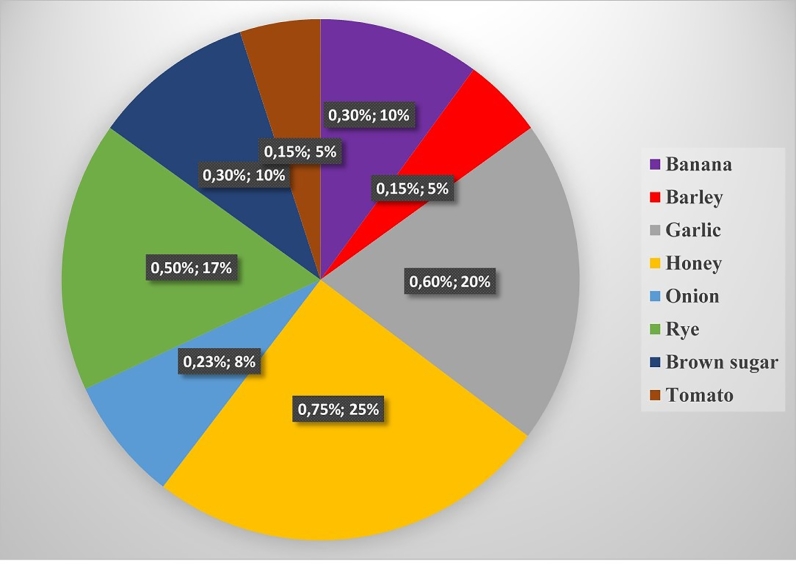
FOS concentration in some natural foods mentioned according to the data of environmental protection agency dietary risk (Sangeetha, 2003).

Fructooligosaccharides are non-digestible oligosaccharides of fructose consisting of a glucose unit (G) connected with fructosyl units (F) at *β*-(2 1) position of sucrose [22, 63, 64]. In addition, they consist of 1-kestose (GF_2_) (Fig. 2a), nystose (GF_3_) (Fig. 2b), and 1-*β*-d-fructofuranosyl nystose (GF_4_) (Fig. 2c), which have 1–3 fructose units’ bond to the *β*-(2,1) position of sucrose (Fig. 2) [31, 65, 66]. FOS derived from sucrose are produced in many higher plants as reserve carbohydrates. These plants include asparagus, garlic, chicory, sugar beet, Jerusalem artichoke, onion, wheat, and tomatoes while some are found in trace amounts in edible fruits like banana (Fig. 3). FOSs are short-chain carbohydrates, which are not digested in the upper part of the gastrointestinal tract; they are also referred to as non-digestible oligosaccharides [15, 67]. The linkage type between their monosaccharide residues distinguishes FOSs.

FOS can be produced using three methods: extraction from inulin-rich plant material, enzymatic synthesis of sucrose, or degradation of inulin by enzyme hydrolysis [68], [69], [70]. However, the majority of FOS, which are food ingredients, are synthesized through enzymatic degradation of inulin from plant polysaccharides or synthesized from sucrose by fructosyltransferase activity [71]. FOS is synthesized in large-scale industrial production by a wide array of enzymes such as inulinases and fructosyltransferases [72, 73]. The various microbial and plant sources of FOS are in Table 2.

Synthesis of FOS occurs through the catalytic action of transfructosylating enzymes, which are classified into two categories: Ftase *β*-d-fructofuranosidase (EC 3.2.1.26) and fructosyltransferases (Ftase, EC 2.4.1.9) [23, 74]. Ftases possess both hydrolytic and transfructosylating activity, as it releases glucose molecule from sucrose by cleaving the *β*−1, 2-glycosidic linkage, thereby shifting the fructosyl group to sucrose, forming FOS products [73]. Ftases exhibit high transfructosylating activity by catalyzing the transfer of fructosyl moiety from one sucrose molecule to another to produce higher FOS units as major products [23]. These enzymes occur in many higher plants such as *Cichorium intybus* and *Helianthus tuberosus* that produce high levels of Ftase such as sucrose fructosyltransferase (1-SST, EC.2.4.1.99) and fructose 1-fructosyltransferase (1-FFT, EC 2.4.1.100) [75]. Fungi including *Aspergillus niger* ATCC 20,611, *Aspergillus niger* AN 166, *Aspergillus foetidus, Aspergillus oryzae* CFR 202, and *Aureobasidium pullulans* CFR 77 have been largely documented to contain enzymes with both hydrolytic and transfructosylating activities [17]. Bacterial strains have also been reported to produce Ftase for FOS production, but only few species have been mentioned, which include *Bacillus macerans, Lactobacillus reutri, Streptococcus mutans* and *Zymomonas mobilis* [17, [76], [77], [78], [79], [80]].

Fructooligosaccharides are natural food products with beneficial health effects to the human colon by selectively stimulating the proliferation of *Bifidobacteria* and *Lactobacilli* while concurrently suppressing the growth of potentially pathogenic microbiota such as *Clostridia* [8, 15]. It is for these reasons that, FOSs have received particular attention as biofunctional food products. FOS has generated a great demand in the global food market and is generally regarded as safe (GRAS) [81]. Due to these properties and functionalities as alimentary canal additives, suitability for diabetics; non-cariogenic and nutraceutical compounds, they are termed prebiotics [21, [82], [83], [84], [85]].

Prebiotics are compounds that selectively stimulate proliferation of gut microbiota in the colon by inhibiting pathogenic microbes; protonation of potentially toxic ammonia and amines; diminution of total cholesterol in the blood; relieving constipation, triglyceride and phospholipids [86]. The human colon is one of the most colonized and metabolically active organs in the human body. It presents different bacterial compositions and variability, largely due to different physicochemical conditions such as favorable pH, slow transit time, and nutrient availability in the gut [86]. The human digestive system lacks the necessary enzyme to hydrolyze *β*-glycosidic linkages of sugars consumed and as such, non-digestible oligosaccharides can ferment these sugars, creating a prebiotic effect. Prebiotics also display secondary functions including mineral absorption, synthesis of vitamin B-complex, immune system activation, and non-cariogenecity [87]. The human gut ferments a range of carbohydrates that pass the small intestines and are available for fermentation in the colon [84].

### 3.3 Chemical structure of fructooligosaccharides

Fructo-oligosaccharides are inulin-derived, short-chain oligosaccharides, containing d-fructose of linear polymers and oligomers joined together by *β*-(1,2) linkages [88]. A glucose molecule typically resides at the end of each fructose chain, where it's linked by an *α*-(1,2) bond as in sucrose [89]. Inulin is a highly polymerized fructan with a chain length ranging from 2- 60 units and a DP of 25 with molecular distribution ranging from 11 to 60 [90]. They are depicted by the formula GF_n_ and constitute a series of homologous oligosaccharides gleaned from sucrose. In addition, FOSs are members of the fructan group, consisting of a general glucose unit linked to several fructose units. Fructans present in nature can be distinguished based on glycosidic linkages, where fructose residues are linked together [88]. They can be divided into three: the first group is inulin, where fructose units are linked through *β*-(2,1) bond; the second group are levans, which are linear fructans, and the fructose units are linked *via* a *β*-(2,6) bond; the third group is graminian fructan, which is of mixed type, consisting of both *β*-(2,1) and *β*-(2,6) linkages between fructose units [91].

Chain length or DP has a vital role in inulin functionalities. Functional attributes of inulin and oligofructose is attributed to their chain length. Inulin has a longer chain length than oligofructose, which makes it less soluble and forms inulin microcrystals when sheared in water or milk [92]. Oligofructose is a fructose oligosaccharide containing 2–10 monosaccharide residues connected by glycosidic linkages [71]. Oligofructose is reported to have a shorter chain oligomer and possesses similar functional properties to glucose syrup or sugar [93]. Its solubility is higher than sucrose and accounts for 30–50% of sugars. Oligofructose has numerous nutritional properties such as providing crispiness to low-fat cookies, acts as a binder in nutritional or granola bars [94]. Since inulin and oligofructose have desirable functional properties, they are used together and offer dietary fiber effects, leading to reduced caloric effects in foods when compared to typical carbohydrates because they possess *β*-(2,1) bonds linking fructose molecule [92].

### 3.4 Fermentative production of fructooligosaccharides

Studies on fermentation parameters are critical to obtaining maximum yields of FOS. The two main methods documented so far for the production of FOS include submerged fermentation (SmF) and solid-state fermentation (SSF) [95]. Numerous studies have been reported on FOS production using submerged fermentation techniques with titres in the range of grams per liter [96, 97]. However, more recently, solid-state fermentation has been preferred as an alternative to submerged fermentation for the production of oligosaccharides with higher productivity [98]. For specific applications, SSF is viewed as a desirable approach due to its improvements in reactor designs [99, 100]. However, it's still necessary to establish the optimal conditions under SSF for maximum FOS production [101]. Numerous advantages have been associated with SSF. These include simplicity in operation, which produces high-level products after fermentation [102]. SSF uses low water consumption; requires less sterilization and permits little/no microbial contamination during product formation. In addition, it requires less capital to operate, as it uses simple equipment, less space, and agro-industrial residues as substrates that are converted to bulk chemicals with high volumetric products of high commercial value [31, 103]. The downstream process is easier with reduced stirring and low sterilization. However, there are also drawbacks associated with solid-state fermentation. These include the build-up of temperature, pH, moisture, and substrate concentrations. Since it uses little water, it becomes difficult to control [84]. Moreover, the particle size of the substrate is a variable factor that presents a strong effect during the fermentation process. Since small particle increases surface area between the gas phase and microbes, they can influence the medium by making water and oxygen transfer of nutrients difficult [104]. Furthermore, media optimization is labor intensive and time-consuming for higher yields of FOS [105].

## 4 Inulooligosaccharides production from inulin hydrolysis

With the increasing demand for nutritional food, significant attention is being paid to functional foods. Aside from the basic nutrition, the functionality of food with high production value and nutraceutical effect is in great demand [21, 106]. These predominant reasons have led to the production of IOS, which is a class of prebiotic. Overwhelming consumer consciousness for healthier food has heightened the fast growth of the functional food market for IOS [107].

**Fig. 4:**
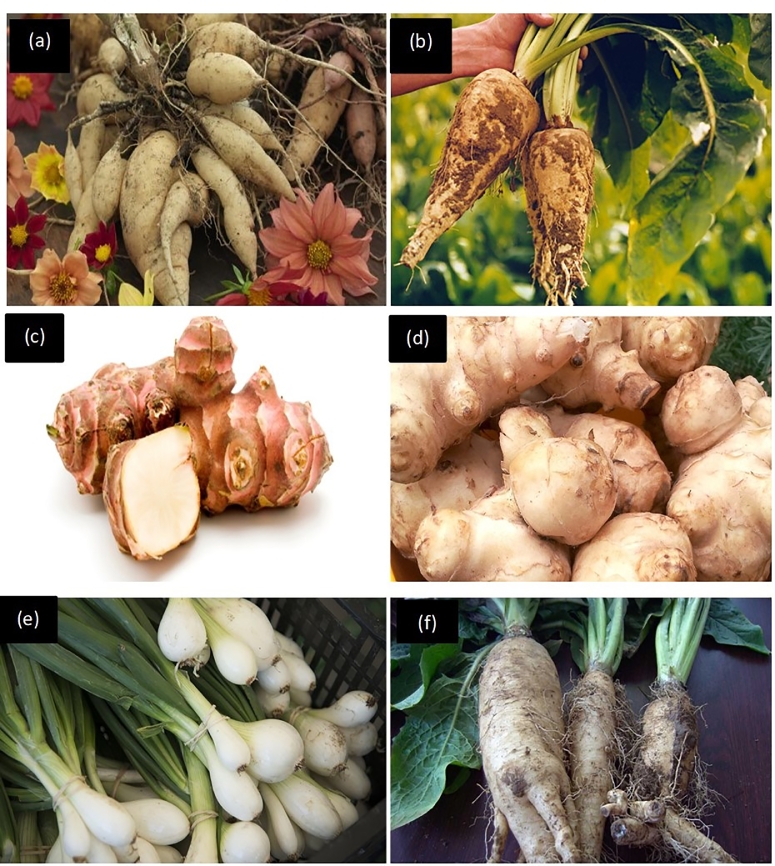
Photographs of inulin producing plants **a** and **b** chicory flowery plants and its storage roots (*Cichorium intybus*), **c, d and f** Jerusalem artichoke (*Helianthus tuberosus*), and **e** onions.

**Fig. 5:**
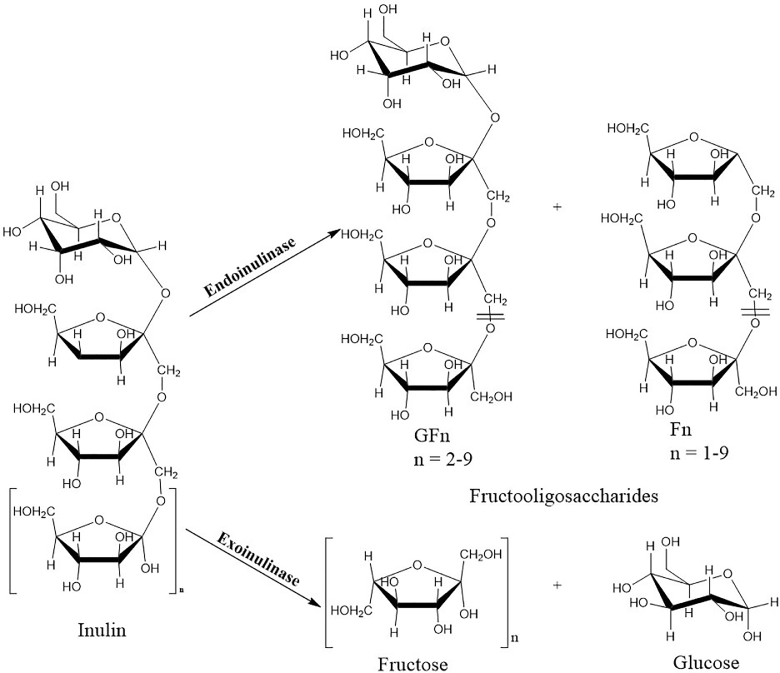
Degradation pattern of inulinase on inulin (Adapted from (Roberfroid et al., 1998)(Singh et al., 2017; Singh & Singh, 2010).

Inulin as a substrate can be regarded as a promising source for inulooligosaccharide production [108]. IOSs produced from inulin hydrolysis are reported to have homogeneous biochemical and physiological functions [109, 110]. Inulin with high DP has shown good prebiotic potential [108, 111]. This is due to its resistance to digestion by the gut enzymes because of the presence of fructose in their *β*-configuration [112]. However, the DP varies from different plant species, age of plant, climatic conditions, harvesting periods, and inulin-rich plant organic material [108]. Inulin serves as a reserve carbohydrate of vegetable and plant polysaccharides. It is found in the underground roots and tubers of dahlia *(Dahlia pinnata*), chicory (*Cichorium intybus*), Jerusalem artichoke (*Helianthus tuberosus*), asparagus (*Asparagus racemosus*) and dandelion (*Taraxacum officinale*) as illustrated in (Fig. 4) [113]. Inulin consists of linear chains of *β*-(2–1)-d-fructosyl fructose links terminated by a glucose residue *via* a sucrose-type linkage at the reducing end [107, 114]. Regioselective reaction and mode of action of inulin with inulinases release fructose units or inulooligosaccharides [115, 116]. (Fig. 5). There are several types of fructans such as inulin, levan, phlein, kestoses, kesto-*n*-oses and graminian [21]. However, inulin fructan is a potential substrate for the production of ultra-high fructose syrup (UHFS). The partial hydrolysis of inulin using endoinulinases yields oligofructose with an average DP of 4. Lower DP oligosaccharide is composed of inulobiose (F2), inulotriose (F3), inulotetraose (F4), inulopentaose (F5) inulohexose (F6) and prebiotic IOS [22, 113, 117].

**Fig. 6:**
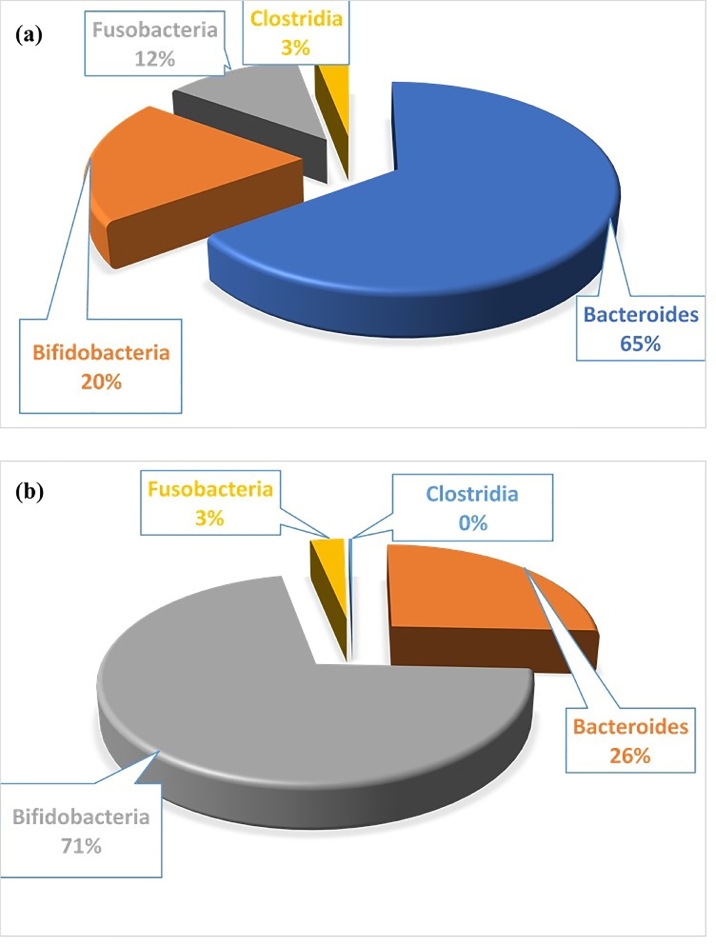
Prevalence of pathogenic microbes (a) before and (b) after the uptake of inulin. The proliferation of *Bifidobacteria* after inulin intake showing the prebiotic effect of inulo-oligosaccharide.

Inulin-type fructans have desirable properties similar to FOS. These include high sweetness intensity, as they are third sweeter as sucrose and this feature is important in foods restricted with sucrose [118]. Secondly, IOS has low calories levels, which are rarely absorbed by the upper part of the gut and consequently are not used up as an energy source, making them safe for consumption by diabetics [21]. Third, IOSs are non-cariogenic, that is, they are unused by *Streptococcus mutans* to form acids and β-glucan, which is insoluble and a major cause of dental caries [70]. Fourth, inulin-type fructans act as prebiotics since they promote the growth of *Bifidobacteria* while concomitantly suppressing the growth of potentially putrefactive microbes in the digestive tract [21, 119]. These properties improve gut functions. The evaluation of gut microflora before and after inulin intakes is illustrated in Fig. 6.

## 5 Enzyme-mediated production of inulooligosaccharides and fructooligosaccharides

Complex carbohydrates are difficult to synthesize hence require alternative methods that can degrade polysaccharides to maximize yields. Inulin hydrolysis has been employed in the production of syrup with high fructose concentration [107]. The reaction was carried out using an acid catalyst and was found to present several shortcomings including high processing temperature, leading to high energy consumption, inulin precipitation, and microbial contamination [120]. In addition, by-products with no sweetening capabilities, resulting in an overall decrease in yields were also reported. Several other drawbacks of chemical hydrolysis include extended time for refluxing, found to require acid-resistant equipment [21]. Moreover, the processes are tedious, as they involve protection, deprotection, and activation strategies to control the stereochemistry and regioselectivity of the resulting oligosaccharide, which is undesirable and unrealistic for large-scale production [121, 122]. In addition, the chemical method requires the use of hazardous & expensive chemicals and results in low yields and high production costs. Due to the aforementioned challenges, the application of microbial enzymes for oligosaccharide production is recognized as an attractive strategy [27, 123].

Application of enzyme-based approach for catalytic production of oligosaccharides has been applied as an alternative technique to acid and chemical hydrolysis due to its simplicity in preparation, rapidity, and reproducibility in mild reaction conditions and easy separation of products [124]. Enzymatic approach consumes less energy, as it requires low temperatures, produce less toxins and pollutant to the environment, and produces fewer emissions and by-products [21, 120]. Enzymatic method has been demonstrated as a suitable approach for industrial oligosaccharide production [21, 125]. For instance, the use of inulinase has been reported to produce 95% pure fructose [126, 127]. Other products include IOS mixture, consisting of inulotriose, inulotetraose, inulobiose, inulopentaose, inulohexose and minimal glucose [21].

## 6 Enzymes used for oligosaccharides’ production

Fructo-oligosaccharide is produced by the transfer of fructose residues to sucrose molecules by the action of fructosyltransferase (E.C.2.4.1.9), β-fructofuranosidase (E.C.3.2.1.26), or inulinase (Table 3) [27, 128]. Inulinases are divided into two subclasses due to their mode of action: exoinulinases (EC: 3.2.2.80), which cleaves fructose from the non-reducing sugar end of inulin through hydrolysis and is mainly used in the synthesis of ultra-high fructose syrup [129]. Endoinulinases (EC: 3.2.1.7) hydrolyses inulin into IOS [114]. IOS produced from inulin possesses corresponding physiological functions to FOS with variations in DP [130]. Numerous microorganisms including *Aspergillus niger, Aspergillus ficuum, Arthrobacter* sp, *Penicillium purpurogenum, Bacillus macerans* and *Streptococcus mutans* are sources of endoinulinases [78, 80]. Moulds are the most prominent groups producing endoinulinases [131]. Interestingly, few fungal species have both exo and endoinulinase properties [108].

## 6.1 Fungal fructosyltransferases

Fungal Ftases have a molecular mass ranging from 180,000 to 600,000 and are homopolymers with 2–6 monomers [132]. Fructofuranosidase isolated from *Aspergillus oryzae* is a monomer with a molecular weight of 87,000 - 89,000 [28, 84]. Several studies on transfructosylating enzymes secreted by *Aspergillus* and *Aureobasidium* produced maximum yields of FOS. The enzyme displayed both hydrolytic and transferase activity [95, 133]. Yoshikawa et al. (2006) reported fructosyltransferase from the cell wall of *Aureobasidium pullulans* with high transferase activity with the lowest Km value for sucrose 139 mM [134]. In fungi, Ftase 1 plays a major role in FOS formation while Ftase IV has strong hydrolytic action that may degrade FOS [84]. Several fungi species such as *Aspergillus, Aureobasidium,* and *Penicillium* are known to produce both intracellular and extracellular β-fructofuranosidase and fructosyltransferase [133, [135], [136], [137], [138], [139]]. Predominantly, *Aspergillus* species have received particular interest in microbial FOS production [140, 141]. *Aspergillus niger* and *Aspergillus oryzae* have been exploited for enzyme production since they have GRAS status [132]. Other fungi such as *Penicillium rugulosum* and *Aspergillus phoenicis* CBS 294.80, which secrete a thermostable inulinase for industrial fructose production also produce a sucrose-1^F^-fructosyltransferase, SFT (E.C 2.4.1.99) [142, 143]. Fungal ftases have been the focal point, as numerous studies on industrial biotechnology have described the isolation and screening of intra or extracellular fructosyltransferase [133, 144]. *Aspergillus japonicus* with other moulds was selected after a screening exercise for the ability to produce transferase [145]. In addition, Madlov et al. (2000) selected *Aspergillus pullulans* and *Aspergillus niger* for their potential to produce fructosyltransferase [146]. Furthermore, Fernandez et al. (2007) screened seventeen filamentous fungi grown in batch cultures and compared their ability to produce β-fructofuranosidase and fructosyltransferase [147]. The findings revealed three strains of *Aspergillus niger* ATTC 20,611, IPT-615 and *Aspergillus oryzae* IPT-301 as good candidates for industrial fructosyltransferase production.

Screening of new fungal isolates is always a difficult procedure due to a number of evaluations. However, numerous reports still exist on screening fungi for biotechnological application. A presumptive and indirect colorimetric plate assay was employed for screening of a filamentous fungus for transfructosylation ability [148]. The method was carried out to determine the simultaneous release of fructose and glucose from sucrose biotransformation. A glucose oxidase-peroxidase reaction using phenol and 4-aminoantipyrine was used for glucose determination. Fructose dehydrogenase oxidation in the presence of tetrazolium salt was used for fructose determination. The formation of a pink halo revealed the presence of glucose while blue halo formation confirmed the presence of fructose and transfructosylation activity. Other studies on screening fungal and yeast species for fructosyltransferase production have also been reported, as they are a more feasible and economic source of biocatalytic enzymes [18, 87, [149], [150], [151]]. Based on these evaluations, fungal fructosyltransferase is more desirable than plant and bacterial fructosyltransferase for large-scale production of FOS. This is due to their physicochemical characteristics including minimal loss of enzyme activity, by-product inhibition, and low molecular weight, which allows easier separation of the biocatalyst from the product.

## 6.2 Bacterial fructosyltransferases

FOS-producing enzymes are rarely secreted among bacterial species, but notwithstanding some strains of bacteria have been reported to be inulinase producers [31]. A study by Hicke et al. (1999) reported *Streptococcus mutans* as the only known source of bacterial inulinase [152]. In earlier studies, cloning and sequencing of the *β*-d-fructosyltransferase was reported from *Streptococcus salivarius*. The recombinant fructosyltransferase was expressed in *Escherichia coli* and later purified to homogeneity [153]. The enzyme catalysed the transfer of fructosyl moiety of sucrose to multiple receptors including glucose, water, and unhydrolysed sucrose *via* the Ping Pong mechanism of fructosyl-enzyme intermediate [154, 155]. A transfructosylating enzyme from *Bacillus macerans* EG-6 produced FOS with a yield of 33% in the presence of 50% sucrose as substrate [80]. A novel strain of *Bacillus licheniformis* was reported to be capable of producing FOS and a polysaccharide-type levan [156, 157]. An ethanol-producing bacteria strain of *Zymomonas mobilis* has been reported to produce levansucrase, capable of producing FOS and levan [158]. Levansucrases are fructosyltransferases belonging to the family 68 of glycoside hydrolases, which catalyzes FOS formation and synthesis of *β*-(2,6) levan [156]. In this study, extracellular levansucrase along with levan as the supernatant was used as biocatalyst in FOS sugar syrup. FOS yield of 24- 34% was obtained, comprising of 1-kestose, 6-kestose, neokestose and nystose [31]. Glucose which formed as a by-product during FOS production was found to inhibit transfructosylation reaction along with ethanol (7%) in sucrose syrup [159]. The fructan syrup group showed prebiotic characteristics. In another study, a strain of *Lactobacillus reutri* 121 was reported to produce 10 g/L FOS (95% 1-kestose and 5% nystose) in the supernatant when grown on sucrose medium as a carbon source. Fructosyltransferase obtained from the strain when incubated at 17 h with sucrose also produced FOS and 0.8 g/l inulin [160, 161]. A new study reported levansucrase gene (LmLEVS) cloned from *Leuconostoc mesenteroides* MTCC 10,508. The heterologous expression and purification of the truncated (TrLmLEVS) gene, lacking the N-terminal signal peptide, was performed in *E. coli*. The recombinant enzyme (TrLmLEVS) was physico-kinetically characterized using sucrose as substrate and the physiochemical and kinetic properties of the levansucrase gene from *L. mesenteroides* MTCC10508 (TrLmLEVS) characterized. The study demonstrated the synthesis of fructo-oligosaccharides and levan from sucrose by the catalytic action of TrLmLEVS [212]. A similar study described the cloning, heterologous expression, and characterization of the levansucrase gene *Ca-SacB* from *Clostridium acetobutylicum*, which laid the foundation for further modification of this enzyme for more efficient production of fructan from transfructosylation by *Ca-SacB* [213]. Furthermore, the effect of ten commercially available oligosaccharides was tested *in vitro* on the growth of *Lactobacillus* strains including *Lactobacillus reutri* C 16, *Lactobacillus salivarious* I 24*, Lactobacillus gallinarum* I 16 and *Lactobacillus bevis* I 25. From the investigation, oligosaccharide utilization varied among the *Lactobacillus* strains. Good growth of *Lactobacillus* was supported by isomaltooligosaccharides (IMO), GOS, and FOS. The results indicate that oligosaccharide utilization by *Lactobacillus* could be both strain and substrate-specific [83].

## 6.3 Microbial exoinulinases

Inulin is a polyfructan containing linear β−2,1 linked polyfructose chain and is considered to be the most suitable substrate for enzyme production [129]. It is also considered a renewable source of raw material in fructose syrup manufacturing and FOS production [162]. It is insoluble in water due to variations in chain length elongation and molecular weight, which varies between 3500 - 5500. Microbial inulinase (2,1-*β*-d-fructan fructohydrolase EC, 3.2.1.80) catalyzes inulin hydrolysis by cleaving d-fructose from non-reducing sugar (*β*−2,1) end of inulin [129]. Microbes involved in exoinulinase production include species of *Penicillium, Aspergillus, Kluyveromyces, Sporotrichum, Cryptococcus, Pichia, Cladosporium, Bacillus, Pseudomonas, Xanthomonas, Sporotrichum* and *Candida* [13, 163, 164].

### 6.4 Microbial endoinulinases

Microbial endoinulinases (2,1-*β-*d-fructan-fructan hydrolase, EC3.2.1.7) act on the internal linkage of inulin randomly to form intermediates such as inulotriose, inulotetraose and inulopentaose [21]. It is observed that similarities exist between exoinulinases and endoinulinases and this makes it difficult to separate by conventional methods. However, Native-polyacrylamide gel electrophoresis has been proposed as an efficient tool to separate enzymes showing similar characteristics [165]. Endoinulinase that is free from invertase or exoinulinase activity has been investigated and reported to hydrolyze inulin internal linkages and thus produce several oligosaccharides which are soluble dietary fiber with low caloric value [130].

## 7 Potential health benefits of oligosaccharides

### 7.1 Prebiotics

**Fig. 7:**
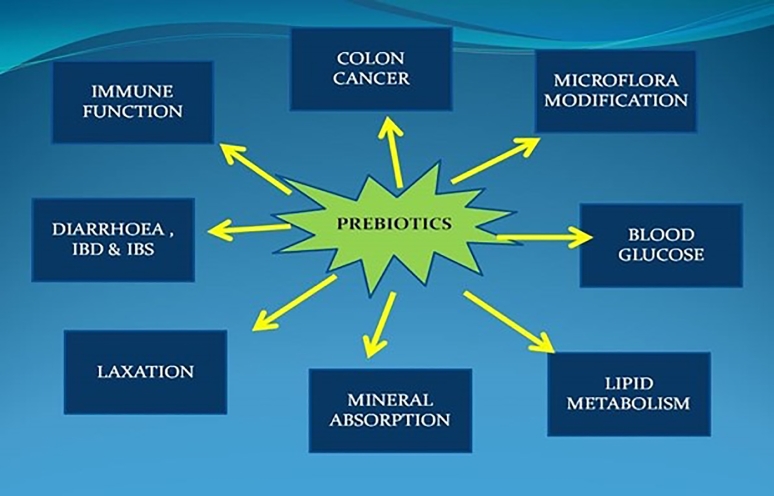
Beneficial impacts of *Bifidobacteria* accumulation in the colon.

Prebiotics are biofunctional food supplements that stimulate selective growth of *Lactobacilli* and *Bifidobacteria* in the gut, leading to improved health [166]. Prebiotics creates an unfavorable environment for harmful invasive pathogens by stimulating *Lactobacilli* and *Bifidobacteria* proliferation [167]. The intestinal bacteria ferment oligosaccharides and produce large compounds of short-chain fatty acid, resulting in acidic conditions in the colon which colonize adhesive sites and secrete bacteriostatic peptides [168]. The prebiotics bacteria survive harsh acidic conditions and are adherent to mucosal walls of the gut by producing organic acids like lactic acid, which are inhibitors of many pathogenic microbes hence improving gut health [169]. Some of the major prebiotic functions are illustrated in (Fig. 7).

### 7.2 Dietary fiber effect

Dietary fibers are plant or carbohydrates analogous that is not easily hydrolyzed in the upper part of the small intestines [170]. They contain edible plant polysaccharides remnants that cannot be easily hydrolyzed by human digestive enzymes (AACC Report 2001). The partial or complete fermentation in the large bowel is crucial in the metabolism of dietary fiber [170]. There is increasing evidence that supplementation of diet with fermentable fiber alters the gut function and structure either by modification or production of gut-derived hormones, which improve glucose homeostasis [171]. It is for this reason that oligosaccharides are associated as part of its identity, as it portrays beneficial physiological characteristics showing similarity with dietary fiber intake [94, 172]. Consumption of dietary fiber provides health benefits to humans, including the bioavailability of minerals and aid in lipid metabolism, thereby reducing risks associated with colon cancer and cardiovascular disease. They can be incorporated into food and drink, as they provide caloric dilution in viscous drinks and diets [71].

### 7.3 Anticancer agent

Diets that contain high proteins, high animal fat concentrations, and low dietary fiber concentrations are linked with colonic cancer [88]. However, oligosaccharides contribute indirectly to colon cancer prevention [55]. Oligofructose administration has been found to decrease genotoxicity [51]. Some bacterial commensals of the colon are carcinogenic and tumor promoters as a result of food metabolism [173]. In the gut, there exist two types of fermentation after ingestion of food proteolytic and saccharolytic enzymes. The latter is more favorable due to metabolic by-products formed such as acetate, SCFAs, propionate, and butyrate [174]. When a model system of the human gut was investigated after feeding galactooligosaccharides, there was a considerable depreciation of nitroreductase, a metabolic activator and carcinogenic substance that decreases indole and isovaleric levels [15]. According to studies done by Kim et al. [122], butyrate has been found to have antitumor characteristics and also up-regulate apoptosis, therefore, contributing to the prevention of colon cancer by promoting cell differentiation [84]. In another study reported by Bali et al. [23], consumption of oligosaccharides was observed to reduce intestinal tumor while increasing the development of lymphoid nodules in the gut-associated lymphoid tissue (GALT). In addition, propionate has chemoprevention properties that induce an anti-inflammatory effect on colon cancer cells [175]. Another study reported the effect of starch administration on human flora-associated rats (HFA), where there was a decrease in ammonia levels and *β*-glucuronidase with high-level caecal butyrate observed. Butyrate which is critical for cancer reduction is not only the primary energy source for colonocytes but also helps to maintain a healthy epithelium. It can also play a large part in cancer prevention. Such interactions include activation of apoptosis, a mechanism that is inactivated in cancer cells that would normally contribute to their death and an increase in the immunogenicity of cancer cells due to an increase in the expression of proteins on the cell surface [176]. Butyrate plays a dual role in maintaining a healthy epithelium as well as provides energy for colonocytes [15]. Furthermore, a decrease in azomethane-induced colorectal cancer in F344 rats when fed on oligofructose diet indicates the anti-cancer potential of the functional food [23].

### 7.4 Mineral absorption

To expand the knowledge of oligosaccharides in improving mineral absorption, several mechanisms have been explained. The consumption of oligosaccharides has been explained in several experimental animals [177, 178]. The dietetic fiber binds to or sequesters minerals, reducing their absorption in the ileum and their arrival in the large intestine [88]. The sequestered minerals along with fermented soluble fiber become available in the colon; high concentrations of SCFAs from colonic fermentation of oligofructose increase solubility of calcium and magnesium ions [24]. The stimulation of magnesium and calcium was also observed in dogs while in adult animals, mineral absorption was stimulated in groups receiving resistant starch or inulin diet. Moreover, there was a significant increase in calcium absorption if there was a combination of the two [179]. Bioavailability of oligosaccharides occurs largely in the colon; this is due to fermentation by commensal microbes [180]. SCFAs decrease luminal pH, leading to an acidic environment favouring solubility of Ca^2+^, Mg^2+^, Fe^2+^ that maintain a homeostatic balance between Fe^2+^and Zn^2+^ [84, 181]. In another study, gastrectomized experimental animals were fed with oligosaccharides. The iron uptake was found to increase, suggesting the significance of the functional food in alleviating anemic conditions. Oligosaccharides uptake was also observed to prevent osteopenia in rats, as calcium ions stored in bones were easily absorbed [23]. Numerous benefits emanate from intestinal calcium and magnesium uptake [6].

### 7.5 Lipid metabolism

Animal studies carried out in mice showed that oligofructan, inulin and non-digestible (but fermentable) oligomer of *β*-d-fructose (obtained by inulin hydrolysis) possess the physiological effect on cholesterol while significantly lowering serum triglyceride levels by decreasing postprandial cholesterolemia and triglyceridemia by 15% and 50%, respectively [182]. The lipogenic decline in enzyme activity and very-low-density lipoprotein (VLDL), which contains the highest amounts of triglycerides particles contribute to this effect [183]. Moreover, FOS fermentation increases propionic acid in intestinal mucosa and in turn reduces levels of triacylglycerol (TAG) and associated hypercholesterolemia LDL and VLDL [23]. In human studies, the use of inulin and oligofructose as food supplements in normal and hyperlipidaemic conditions showed no effects on serum cholesterol or triglyceride. However, three investigations showed a slight reduction in triacylglycerol, while four inspections cholesterol and triacylglycerol lowered significantly [114, 184]. Inulin appears to be more suitable than oligofructose in reducing triglyceridemia while in animal studies, both oligofructose and inulin were equally active [185]. Based on these findings, prebiotics has been shown to affect hepatic lipid metabolism [185]. In a study of diabetic rats, simple carbohydrates were replaced with XOS in their diets and there was a drastic drop in serum cholesterol and TAG in diabetic rats while liver triacylglycerol increased to commensurate levels to that observed in healthy rats [186]. This was attributed to lipogenic enzyme inhibition, resulting from prebiotic fermentation in the gut by the action of propionate [15].

### 7.6 Defense mechanism and immune regulation

Consumption of functional food boosts the immune system [170]. Fermentation of saccharolytic metabolites, resulting from dietary intake is closely associated to be in contact with gut lymphoid tissues which cover the majority of the intestinal immune system [166, 170]. Products of FOS fermentation may modulate the GALT as well as the systemic immune system [171]. A concept of immunity suggested by Saad et al. (2013) showed that innate immune response can be activated by sugar moieties interacting synergistically with innate receptors on the host plasma membrane in dendritic cells and macrophages [185]. Β-glucose oligosaccharide activates immune reactions by binding to macrophages receptors. Orally ingested oligofructose and inulin modulate immune system parameters such as IL- 10 and IFN-γ natural killer cells activity, lymphocyte proliferation, intestinal IgA, and increase polymeric immunoglobulin receptor expression in ileum and colon regulation [170]. Consumption of prebiotics fiber induces bifidogenic microflora as a result of short-chain fatty acid from fiber fermentation and direct contact with cytoplasmic components with immune cells [185].

### 7.7 Antioxidant effect

Antioxidants are natural or synthetic compounds that may delay or prevent oxidative stress caused by physiological oxidants [50]. Conventionally, the antioxidants are divided into two groups: the antioxidants that scavenge directly for active free radicals such as reactive oxygen species (ROS) or reactive nitrogen species (RNS), and antioxidants that inhibit oxidative stress [151, 187]. Free radicals are customarily unsteady and originate from nitrogen (RNS), oxygen (ROS) and, sulfur (Reactive Sulphur Species: RSS) [188]. ROS, RNS, and RSS generation in radical and/or non-radical forms occur in humans and animal cells because of metabolic and physiological processes [189]. Moreover, ROS-induced free radicals from exogenous or endogenous sources can be injurious to the body cell biomolecules, causing impairment to cell functions and oxidative stress or apoptosis [190]. Free radicals have also been implicated in numerous pathologies including cardiovascular complications, neurodegenerative disorders as well as oncogenic complications [191].

Intake of inulin-type oligosaccharides, vitamin C, vitamin E, and carotenoids have been found to have the potential to minimize the harmful effects of reactive species [188]. Dietary intake of antioxidants such as tocopherol, carotenoids, and ascorbate are difficult to disentangle through epidemiological studies from other vital vitamins and ingredients in fruits and vegetables. Nevertheless, several studies published suggest that antioxidants are a major remedy for endogenous damage to DNA, lipids, and proteins [189, 192]. Antioxidants play a key role in immune system activation by causing the proliferation of B and T cells, natural killer cells, and lymphokine-activated killer cells that prevent the body defense mechanism from pathogens [193]. Supplementation with dietary antioxidants counteracts the oxidants thereby boosting the complement system [50].

#### 7.7.1 Antioxidants and cardiovascular disease

Cardiovascular complications are associated with low concentrations of ascorbate, tocopherol, and β-carotene [194]. From cardiovascular studies, oxidative modifications of apolipoproteins B 100 play a key role in the recognition of low-density lipoprotein (LDL). LDL uptake by macrophage receptors leads to foam cell formation and atherosclerotic plaques [195]. Lipid peroxidation has been found to alter reactive products of apolipoprotein B 100, leading to a decrease in net charge, a modification that leads to its recognition by scavenger receptors [196].

Antioxidants have anticancer effects. During cell division, an unpaired lesion of DNA can lead to mutation. Hence, an overriding factor in mutagenesis and carcinogenesis occurs from continuous cell division which is a precursor of tumor cells [197]. An increase in cell division enhances mutagenesis. It is difficult for cancer to emerge in non-dividing cells. Antioxidant intake can decrease carcinogenesis and mutagenesis in two ways: by decreasing oxidative DNA damage and by decreasing cell division [193].

#### 7.7.2 Antioxidants and cataracts

Most common ophthalmology procedures involve cataract removal. Taylor and Allen (1992) investigated the impressive evidence that cataracts have oxidative etiology and dietary antioxidants can prevent their formation in humans [198]. Findings from five epidemiological studies assessed the effect of dietary antioxidants on cataracts and showed the deterrent effect of ascorbate, tocopherol, and carotenoids. Those individuals placed on tocopherol or ascorbate supplements daily active ingredient vitamin E succinate (VES)-grafted-chitosan oligosaccharide had about one-third risk of developing cataracts [199], [200], [201], [202], [203]. Other factors causing oxidative stress include cigarette smoking and radiation [204]. The eye protein shows an increased level of methionine sulfoxide, and more than 60% oxidation occurs on methionine residues, causing cataracts. Decrease or abstinence from smoking and increase in dietary consumption of antioxidants is a promising strategy to reduce cataracts.

Various experimental models have been used to analyze the antioxidant potential of free radical scavengers and inhibitors. These models include the 1,1- diphenyl-2-picrylhydrazyl (DPPH) method, which is used to evaluate the free radical scavenging ability of natural antioxidants in food and beverages [151, 205, 206]. Ferric reducing antioxidant power assay (FRAP) is based on the reduction of Fe^3+^-TPTZ complex to the ferrous form at low pH. This reduction is monitored by measuring the absorption spectrophotometrically at 593 nm [207, 208]. Moreover, Ojwach et al. (2020) reported a nitric oxide assay (NO) using Griess reagent, where a purified FOS reduced NO along with the standard antioxidant in a concentration-dependent manner [50]. Macrophages play a crucial role in the generation of pro-inflammatory molecules including nitric oxide (NO). The inducible nitric oxide synthase enzyme (iNOS) synthesizes NO and the enzyme has been widely characterized to be an inducer of both chronic and acute inflammation [209]. Other assays described also include 2,2- azinobis (3-ethylbenzothiazoline 6-sulfonate) 2,2′-axino-bis-3-ethylbenzothiazoline-6-sulfonic acid (ABTS), oxygen radical absorption capacity assay (ORAC) [210].

## 7.8 Other applications

Fructo-oligosaccharides employability as functional foods has led to their industrial applications in the food and beverage industry. In beverages, they are used in cocoa, fruit drinks, infant formulas and powdered milk as supplements [88, 166, 177]. In addition, these functional foods are used as probiotics in yoghurt and other milk products to create symbiotic products. Other current applications include puddings and sherbets, desserts such as jellies, confectioneries (chocolate), biscuits, pastries spread (jam), marmalades, and meat products such as fish paste and tofu [56, 211]. Amid the ongoing COVID-19 crisis, the global market for prebiotics in 2020 was estimated at US$4.5 billion and projected to reach a revised size of US$8 billion by 2026, growing at a compound annual growth rate (CAGR) of 9.9% over the analysis period. Inulin, one of the segments analyzed in this review, is projected to record an 8.9% CAGR and reach US$3.3 billion by the end of the analysis period. The U.S. market is estimated to be at $379.8 million by the end of 2022, while the China market is forecast to reach $1.1 billion by 2026. Other reports by GLOBE NEWSWIRE has estimated the market size of FOS to reach $US1.04 billion by 2025, as a result of increased demand for the product as a cost-effective solution for digestion aid. This trend shows the opportunity in research, development and commercialization of oligosaccharides.

## 8 Limitations in upscale production of prebiotic oligosaccharides

The future of FOS in the food and pharmaceutical industries relies on the challenges and trends that can be stated as follows:

Ø The technological and financial feasibility of FOS production must be established.
Ø Microbial enzymes have been regarded as a potential platform to yield FOS with the absence of toxic by-products, however, more insights into the appropriate use of enzymes is required.
Ø A pre-treatment process prior to extraction is a promising method as it increases the extraction yield as highlighted in this review.
Ø Challenges and opportunities exist in exploring improved knowledge of the synbiotic relationships between FOS and colonic microbiota.
Ø It is necessary to study the structure-function relationship and to examine the bioavailability of FOS; as the non-digestible oligosaccharides are mainly metabolized/fermented by the colonic microflora; to produce metabolites/by-products that exert beneficial biological effects.
Ø The current scenario of FOS as functional food ingredients in food applications is limited to *in vitro* laboratory-scale experiments and needs to be scaled up.

## 9 Conclusions and future direction

Biofunctional properties and health benefits of oligosaccharides have increased the importance of bioprospecting for novel, cheap and renewable bioresources for their production. FOS are synthesized *in vitro* from precursors such as sucrose using fructosyltransferase secreted by coprophilous fungi. Furthermore, IOS can also be produced from the enzymatic hydrolysis of inulin under controlled conditions. However, the main drawback of the production process is low yields of the oligosaccharides, amongst others. Microbial enzymes remain desirable for industrial oligosaccharide production. Moreover, exploration of other techniques including molecular methods to improve the efficiency of the enzymes involved in the synthesis of FOS and IOS is crucial. Further research on genome sequences of dung-inhabiting fungi is currently available. Among them is a classical model of *Podospora anserine*; the release of entire genome sequences will facilitate comprehension of various environmental interactions including their potential for metabolomics studies. Recombinant gene technology should be considered as a predominant promising approach to boost the yield of enzyme production at the industrial level. This application can be used in the cloning and expression of industrial enzymes in an optimized strain for biotechnological exploitation. Genome shuffling is one of such technologies that could be used to improve the specific activity of Ftase by amplifying its genetic diversity. There is a need to study the human gut microbiome beyond *Bifidobacterium* and *Lactobacillus* by evaluating certain areas of nutrition. The nutrigenomics approach using molecular tools could be a starting point towards the future of biofunctional foods

## Funding

Partial funding from the Department of Science and Technology DST-National Research Foundation, Center in Indigenous Knowledge Systems (CIKS), University of KwaZulu-Natal, South Africa.

## Author contributions

J.O conceived and wrote the original draft of the manuscript, review, and editing. AIA was responsible for editing; T.M and SM conceived the study, analysis, investigation, review, editing, supervision and financial resources.

## Ethical approval

This article does not contain any studies with animals performed by any of the authors.

**Table 1:** Investigations of herbivore dung as sources of enzymes.

Source of dung	Aim of the study	Preliminary investigation	References
Giraffe, zebra and impala	To evaluate the feces of wild herbivores in South Africa as a potential source of hydrolytically active microbes	Dung from three indigenous herbivores in Pietermaritzburg, South Africa was sampled. Soil and fecal droppings were measured by triphenyltetrazolium chloride and fluorescein diacetate for hydrolase and dehydrogenase activity respectively. Cellulose, amylase and protease producers were determined by viable plate count on solid agar media containing cellulose, skim milk, starch and Tween 80. Zebra dung displayed the highest hydrolytic activity confirming potential target for new hydrolytic enzyme.	[1]
Cow dung from India	A review on cow dung as a cheap available bioresource.	Cow dung contains high diversity of microbial population. Due to this characteristic, it's feasible to obtain microbial enzymes with potential biocatalytic applications that can be harnessed to produce enzymes from their high microbial diversity. *Bacillus* sp from cow is capable of producing cellulose, carboxymethy cellulose and cellulose.	[2]
Cow dung used as substrate	To produce a protease from dung for enzyme bioprocess	In the study, a halo-tolerant-alkaline protease from *Halomonas* sp. PVI was produced under solid-state fermentation. Cow dung serves as a good substrate for enzyme production of detergent-stable dehairing protease by alkaphilic *B subtilis.* Dehairing process was important as it eliminated use of hazardous sodium sulfide.	[3, 4]
Cow dung	Statistical optimization of fibrinolytic enzyme	Considering its cheap and readily available cow dung was used as substrate for production of fibrinolytic enzyme from *Pseudoalteromonas* sp. under solid-state culture. The newly protease producing *Pseudoalteromonas* sp. has been reported by various researchers as a potential producer of thrombolytic enzyme. Hence, in the reported study it was worthwhile to screen *Pseudoalteromonas* sp. for fibrinolytic enzyme secretion and statistical model of central composite design employed for enzyme production	[5]
Koala feces	Screening dung from koala species for enzymes production	Thirty-seven (37) fungal strains isolated from koala feces were identified by molecular tools of 18S rDNA whereby, they were amplified and sequenced. The enzymes extracted from the fungi were screened for various enzyme production such as xylanase, protease, ligninase and endoglucanase. Using plate agar technique one third of the fungi displayed a halo indicating presence of amylase and tannase activity. Some isolates degraded crystalline cellulose while others displayed lipase activity. It was concluded that koala dung could be harbouring a wide array of biocatalytic enzymes capable of breaking down recalcitrant substrates.	[6]
Cow dung	Investigate potential of enzyme production from herbivore dung	A potent bacteria *Bacillus* sp. Identified by 16S rDNA was isolated from cow dung. On preliminary screening, the strain showed potential to produce a thermotolerant endoglucanase (CMCase). The strain was purified 8.5-fold with a recovery of 39.5% and characterized for different parameters including temperature, the effect of metal ions, chemicals and pH stability. The enzyme in this strain could be applied for bioconversion of lignocellulosic biomass into fermentable sugars.	[7]

**Table 2:** The table below details microbial and plant sources of IOS and FOS synthesizing enzymes.

Fungal source	References	Plant sources	References	Bacterial sources	References
*Aureobasidium pullulans* *Aureobasidium* sp. *Aspergillus oryzae* *Aspergillus japonicas* *Aspergillus niger* *Aspergillus phoenics* *Aspergillus phoenics* *Aspergillus foetidus* *Aspergillus sydowi* *Calviceps purpurea* *Fusarium oxysporum* *Penicillium frequentans* *Penicillium spinulosum* *Phytophthora parasitica* *Penicillium citrinum* *Scopulariopsis brevicaulis* *Saccharomyces cerevisiae*	[8] [9] [10] [11] [12] [13] [14] [15] [16] [17] [18] [19] [20] [21] [21]	*Agave vera cruze* *Agave americana* *Asparagas officinalis* (asparagus roots) *Cichorium intybus* (Chicory) *Allium cepa* *Crinum longifolium* (Sugar beet) *Helianthus tuberosus* (Jerusalem artichoke) *Lactuca sativa* *Lycoris radiate* *Taraxacum officinale*	[22] [23] [11] [24] [12] [24] [13] [25] [26]	*Lactobacillus reuti* *Arthrobacter sp* *Bacillus macerans* *Z. mobilis* *Pseudomonas* sp.	[27] [28] [29] [17] [30]

**Table 3:** A synopsis of studies of microbes used for FOS production produced.

Source of microbe	Enzyme	Optimal condition	Substrate (g/L sucrose)	Yield (%)	Reference
*Aspergillus niger* AS 0023	*β*-fructofuranosidase (EC2.1.4.9) free enzymes Extracellular ftase Intracellular ftase	40 – 60 °C, pH 6.0 −8.5 Sucrose 40 - 70%	500	54	[9]
*Aspergillus japonicus*	*β*-fructofuranosidase (EC 3.2.1.26) free enzymes. Intra and extracellular ftase Extracellular ftase Extracellular ftase	55 °C, pH 5.5, Sucrose 65%	400	55.8	[31]
*Aspergillus oryzae* CFR 202	Fructosyltransferase (EC 2.1.4.9) free enzymes Extracellular ftase	55 °C, pH 5.5, 24 h Sucrose 55%	600	58	[12] [32]
*Penicillium citrum*	Neo-fructosyltransferase free mycelia	50 °C, 40 h - 100 rpm Sucrose 70%	700	55	[33, 34]
*Rhodotorula* sp	Extraxelluar β-fructofuranosidase and fructosyltransferase	72 °C – 75 °C, pH 4.0, 65 °C – 70 °C, 48 h	500	48	[35]
*Z. mobilis*	Levansucrase	24 h	500 – 600	24 – 32	[36]
*Aspergillus* sp N74	Fructosyltransferase (EC 2.1.4.9)	pH 5.5 temp 60 °C at 350 rpm sucrose con 70% w/v	700	57	[37, 38]
*Bacillus macerans* EG-6 *B. macerans* EG-6	Fructosyltransferase (EC 2.4.1.9) free enzymes fructosyltransferase	50 °C, pH 5.0 – 7.0, 100 h 37 °C, pH 6.0, 40 h	500 500	33 GF_4_ (42.3)	[39] [40]
*Aureobasidium pullulans* CFR 77	Fructosyltransferase (EC 2.1.4.9) free enzymes Extracellular ftase	55 °C, pH5.5, 9 – 24 h Sucrose 80%	200	59	[41, 42] [43]
*Aureobasidium pullulans* CCY-27–1–1194	Extracellular and intracellular fructosyltransferase	55 °C, pH 5.5, 48 – 72 h	350	52 – 56	[44]
*Penicillium purpurugenum*	Extracellular and intracellular fructosyltransferase	30 °C, pH 5.5, 720 h	10	58	[45]
*Aspergillus japonicus*	*β*-fructofuranosidase	28 °C, pH 5.5, rpm 200, 72 h	150 – 180	55.2	[46]
*Aspergillus aculeatus*	Ftase from commercial enzyme: Pectinex Ultra SP-L	60 °C, pH 5.0 – 7.0, 24 h 60 °C, pH 6.0, 16 h	600 600	60.7 88	[47] [48] [49]
*Penicillium expansum*	*β*-fructofuranosidase	60 °C, pH 5.0 – 6.5,	200	GF_2_ 80%, GF_3_ 19%, GF_4_ 1%	[50]
*Aspergillus foetidus* NRRL 337	Extracellular fructosyltransferase (EC 2.4.1.9)	40 °C – 45 °C, pH 5.0, 120 h	260 – 470	26% – 47%	[51]
*Penicillium citrium* FERM P-15,944	Β-fructofuranosidase	30 °C, pH 4.0, 100 rpm, 72 h	100	57	[52]

## Declaration of Competing Interest

The authors declare they have no conflict of interest and have read and approved the manuscript.
